# Clinical value of the caval–aortic index and inferior vena cava diameter for volume assessment in hypernatremic patients

**DOI:** 10.1371/journal.pone.0356486

**Published:** 2026-08-27

**Authors:** Tuba Şafak, Ahmet Burak Erdem

**Affiliations:** Department of Emergency Medicine, Ankara Etlik City Hospital, Ankara, Turkey; Ibaraki Children’s Hospital: Ibaraki Kenritsu Kodomo Byoin, JAPAN

## Abstract

**Background:**

Accurate assessment of intravascular volume status in hypernatremic patients presenting to the emergency department is often challenging due to advanced age, altered mental status, and unreliable physical examination. Bedside ultrasonography may provide rapid, noninvasive support for early volume evaluation. This study aimed to assess the clinical utility of inferior vena cava (IVC) diameter and the IVC-to-aorta (IVC/Ao) ratio in relation to early sodium response in patients with hypernatremia.

**Methods:**

This prospective observational study included 60 adult patients with hypernatremia presenting to a tertiary emergency department. Ultrasonographic measurements (IVC diameter, aortic diameter, and IVC/Ao ratio) and manometric measurements (central and peripheral venous pressures) were obtained before fluid resuscitation and repeated at the third hour of treatment. Early sodium response was exploratorily assessed using the 3-hour change in serum sodium (ΔNa_1_). Receiver operating characteristic analysis was used to determine the optimal ΔNa_1_ cut-off associated with 30-day mortality.

**Results:**

Patients without early sodium response (n = 42) had significantly higher baseline IVC/Ao ratios than patients with early sodium response (0.74 vs. 0.50, p = 0.037). Ultrasonographic parameters, including baseline and 3-hour IVC diameter and IVC/Ao ratio, demonstrated moderate discriminative performance for predicting early sodium response (AUC range: 0.671–0.683), whereas manometric measurements did not significantly differ between groups. These indices demonstrated high sensitivity but modest specificity for early sodium response.

**Conclusion:**

In hypernatremic patients, bedside ultrasound assessment of IVC diameter and IVC/Ao ratio may provide rapid, complementary physiologic information during early volume evaluation. These measurements should be interpreted alongside clinical and laboratory findings rather than as standalone indicators of treatment success.

## 1. Introduction

Hypernatremia is a common electrolyte disorder defined by a serum sodium concentration exceeding 145 mmol/L and is observed in approximately 1–3% of hospitalized patients [1]. It is associated with particularly high mortality rates—reaching up to 40–60%—among vulnerable populations, including patients of advanced age, those with a high burden of comorbidities, and individuals managed in intensive care units. From a pathophysiological perspective, hypernatremia most commonly results from a relative reduction in total body water compared with electrolyte content and is frequently accompanied by intravascular volume depletion [2,3]. In the emergency department setting, this combination of electrolyte disturbance and volume contraction often coexists with altered mental status, frailty, and limited physiologic reserve, further complicating early clinical evaluation.

The primary therapeutic objective in hypernatremia is to safely reduce serum sodium levels while simultaneously correcting the underlying volume deficit. Treatment success is commonly evaluated based on the rate of sodium correction, as both overly rapid and insufficient correction have been associated with adverse outcomes, including osmotic demyelination syndrome and increased mortality [3,4]. Consequently, accurate assessment of early sodium dynamics and volume status is critical not only for guiding ongoing fluid management but also for prognostic stratification.

Traditionally, intravascular volume status and response to fluid resuscitation have been assessed using invasive manometric measurements such as central venous pressure (CVP). However, increasing evidence has questioned the reliability of CVP in reflecting true volume status, while concerns regarding invasiveness and procedure-related risks have further limited its utility [5]. In this context, non-invasive bedside ultrasonography has gained prominence. Parameters derived from ultrasonographic assessment—particularly inferior vena cava (IVC) diameter and its respiratory variation—have been increasingly used to estimate volume status [6]. More recently, the ratio of IVC diameter to aortic diameter (IVC/Ao index) has been proposed as a static yet physiologically grounded parameter that may provide a more reliable estimate of effective circulating volume, given the relatively fixed caliber of the aorta [7]. By normalizing IVC diameter to a relatively fixed arterial structure, the IVC/Ao ratio may reduce inter-individual variability related to body size and age.

The primary aim of this study was to compare early ultrasonographic parameters measured at the third hour of treatment (inferior vena cava diameter, aortic diameter, and the IVC/Ao ratio) with manometric measurements (peripheral venous pressure and central venous pressure) in relation to early sodium dynamics in patients presenting to the emergency department with hypernatremia. Early sodium response was evaluated based on short-term changes in serum sodium levels.

As a secondary exploratory objective, we evaluated whether early sodium dynamics and ultrasonographic parameters were associated with 30-day mortality.

## 2. Materials and methods

### 2.1. Study design

This prospective, observational study was conducted in a tertiary care emergency department (ED). Adult patients presenting to the ED with hypernatremia were consecutively enrolled. Patient recruitment began on February 1, 2024, and continued until the predetermined sample size was reached over an approximate one-year period. As this was an observational, non-interventional study without randomization or experimental treatment assignment, prospective clinical trial registration was not required. The study was conducted in accordance with the Declaration of Helsinki and approved by the Ethics Committee of Ankara Etlik City Hospital (AEŞH-BADEK-2024–031).

### 2.2. Participant selection

**Fig 1:**
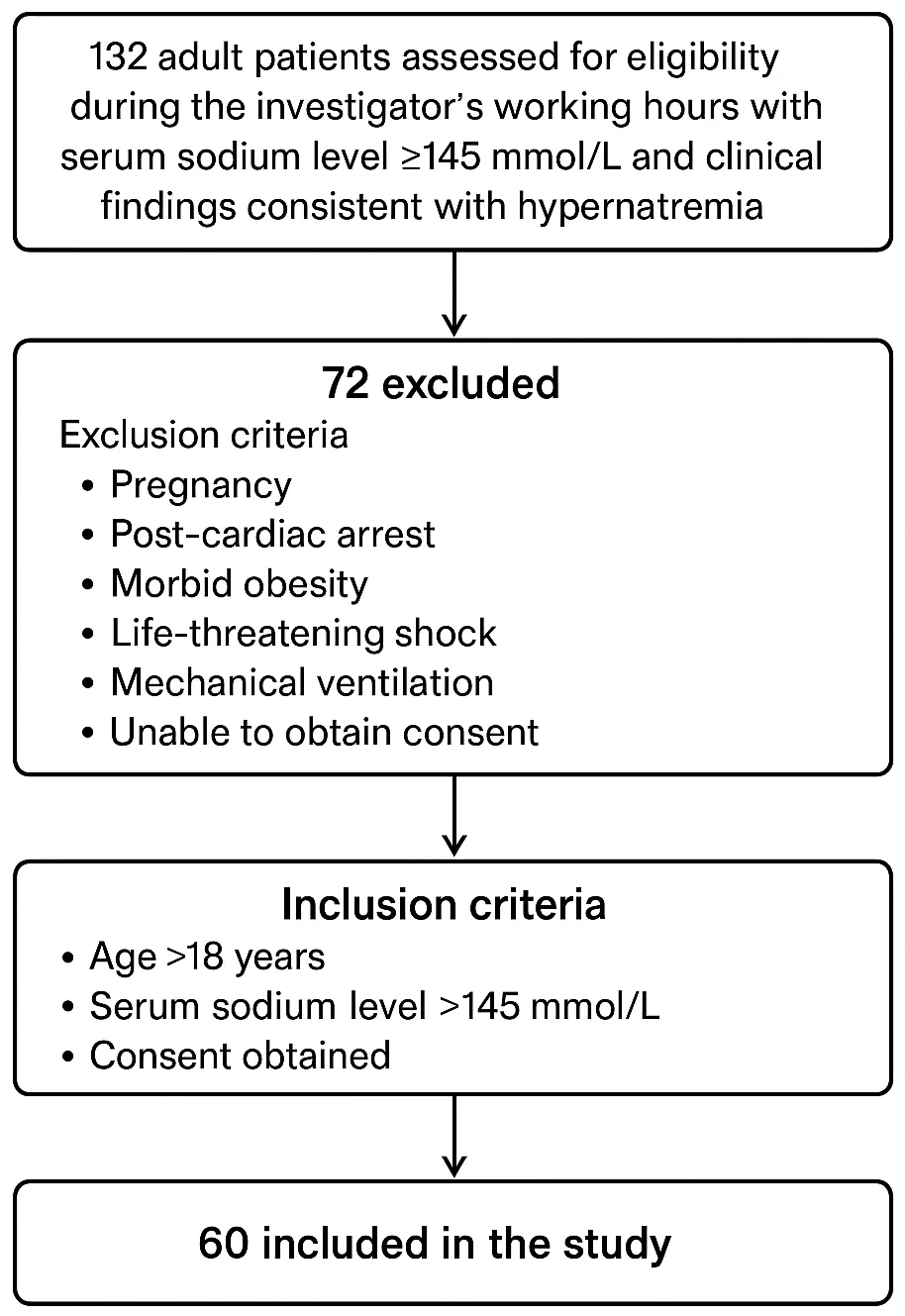
Flowchart of patient screening, exclusion, and enrollment.

During the study period, 132 adult patients presenting to the emergency department during the investigator’s working hours with a serum sodium level ≥145 mmol/L and clinical findings consistent with hypernatremia were screened for eligibility. This approach was chosen due to investigator availability and is acknowledged as a potential source of selection bias. Of these, 72 patients were excluded due to life-threatening shock requiring immediate intervention, the need for mechanical ventilation during follow-up, initiation of fluid therapy prior to baseline ultrasonographic and manometric measurements, morbid obesity, or refusal to provide informed consent. The patient screening, exclusion, and enrollment process is summarized in the study flowchart (Fig 1).

The remaining 60 patients who met the inclusion criteria and underwent central venous catheterization were enrolled in the study. All enrolled patients received intravenous fluid resuscitation calculated according to estimated fluid deficit and total body water formulas. The type of intravenous fluid was selected based on the anticipated sodium reduction calculated using the Adrogué–Madias formula. Importantly, all treatment decisions, including fluid type and administration strategy, were made at the discretion of the primary treating physician, and no therapeutic intervention was directed by the study investigators. Written informed consent was obtained from each patient or their legal representative prior to participation.

This predominantly elderly and multimorbid cohort reflects the typical clinical population in whom hypernatremia is most frequently encountered in emergency department practice.

### 2.3. Data collection and manometric and ultrasonographic measurements

Patients’ demographic characteristics, including age, sex, comorbidities, and underlying etiology of hypernatremia, were recorded using a standardized study form based on direct patient interviews and review of electronic medical records. Vital signs, laboratory parameters, emergency department outcomes, and 30-day mortality data were similarly obtained from electronic medical records.

For manometric measurements, each participant was placed in the supine position. A saline-filled, air-free infusion set was connected to a central venous catheter inserted into the femoral vein, and catheter patency was confirmed by unobstructed fluid flow. The intersection of the fourth intercostal space and the midaxillary line (phlebostatic axis) was used as the reference point for central venous pressure measurement in the supine position [8]. The vertical distance between the stabilized fluid level within the infusion set and the level of the right atrium was measured in centimeters and recorded as the central venous pressure. The same procedure was applied using a peripheral venous catheter placed in the antecubital region to obtain peripheral venous pressure measurements.

Ultrasonographic measurements were performed by a single emergency medicine specialist with formal ultrasound training to minimize interobserver variability. Imaging was conducted using a LOGIQ P9® ultrasound system (GE Healthcare, Chicago, IL, USA) with a convex probe. The inferior vena cava (IVC) and abdominal aorta (AA) were initially identified in the transverse plane and confirmed using Doppler ultrasonography. Subsequently, a longitudinal view was obtained through the subxiphoid hepatic window in B-mode. The IVC was visualized approximately 2 cm distal to its junction with the right atrium, and the probe was angled laterally to record the maximal IVC diameter. Inner-to-inner wall measurements were obtained over three respiratory cycles, and the largest value was recorded.

Abdominal aortic measurements were performed with the patient in the supine position. After identification in the transverse plane along the mid-abdominal line using Doppler ultrasonography, the probe was rotated to obtain a longitudinal view, and an inner-to-inner wall diameter was measured approximately 10 mm proximal to the celiac trunk. The IVC/Ao index was calculated as the ratio of the maximal IVC diameter to the aortic diameter.

At the third hour of treatment, both manometric and ultrasonographic measurements were repeated to assess changes in volume status and early sodium response. During follow-up, the total volume of administered intravenous fluids and urine output were continuously monitored and recorded in the case report form.

### 2.4. Sample size

Based on the planned analyses and previously published studies, an a priori sample size calculation indicated that a minimum of 54 participants would be required to detect a moderate effect size. The calculation assumed an effect size of 0.5, a two-sided α level of 0.05, and a statistical power of 80% [9]. Sample size estimation was performed using G*Power software (version 3.1).

### 2.5. Outcomes

The primary outcome of the study was the association between ultrasonographic parameters and early sodium dynamics in patients with hypernatremia. Changes in serum sodium levels were calculated between baseline and the third hour of treatment (ΔNa_1_), as well as between the third and sixth hours (ΔNa_2_). Ultrasonographic measurements were limited to baseline and the third hour; therefore, ΔNa_2_ was evaluated for descriptive and follow-up purposes only and was not incorporated into outcome classification.

The association between early sodium change (ΔNa_1_) and 30-day mortality was assessed using receiver operating characteristic (ROC) curve analysis, and the optimal cut-off value was determined using the Youden index. Based on this cut-off, patients were exploratorily categorized into two groups according to their early sodium response. Patients with a sodium reduction exceeding the identified cut-off were classified as the “Response” group according to early sodium dynamics, whereas those with a sodium reduction below the cut-off were classified as the “No Response” group.

Importantly, this classification does not represent a standardized clinical definition of fluid responsiveness or overall clinical recovery, but rather reflects an exploratory, outcome-oriented biochemical stratification based on early sodium changes observed in this cohort.

### 2.6. Statistical analysis

All statistical analyses were performed using Jamovi statistical software (version 2.6.26, Sydney, Australia). Descriptive statistics were expressed as median and interquartile range (IQR) for continuous variables with non-normal distribution and as frequency and percentage (%) for categorical variables.

The distribution of continuous variables was assessed using visual methods (histograms and Q–Q plots) and analytical testing with the Shapiro–Wilk test. As most continuous variables did not meet normality assumptions, non-parametric statistical methods were applied.

Within-patient comparisons of pre- and post-fluid resuscitation measurements (central venous pressure, inferior vena cava diameter, and IVC/Ao ratio) were performed using the Wilcoxon signed-rank test. Associations between ultrasonographic parameters (IVC diameter and IVC/Ao ratio) and manometric central venous pressure measurements were evaluated using Spearman’s rank correlation coefficient (ρ). Correlation results were additionally visualized using a correlation matrix presented as a heat map.

Receiver operating characteristic (ROC) curve analysis was used to evaluate the association between early sodium change (ΔNa_1_) and 30-day mortality, and the optimal cut-off value was determined using the Youden index. This ROC-derived threshold was subsequently used to categorize patients according to early sodium response in secondary analyses. In addition, ROC curve analysis was applied to assess the discriminative performance of ultrasonographic parameters (IVC diameter and IVC/Ao ratio) in relation to early sodium response, with area under the curve (AUC) values calculated for each parameter.

A p-value < 0.05 was considered statistically significant for all analyses. Results were presented using appropriate tables and figures.

## 3. Results

### 3.1. Characteristics of study subjects

The study population consisted of 60 patients with a median age of 84.5 years (IQR: 80.0–91.0), of whom 56.7% were female. The cohort had a high burden of comorbidities, with hypertension being the most prevalent condition, followed by coronary artery disease and cerebrovascular disease. Baseline vital signs and laboratory findings, including a median corrected serum sodium level of 159 mmol/L (IQR: 153–169), are presented in Table 1.

**Table 1:** Baseline demographic, clinical, and outcome characteristics of the study population (n = 60).

Parameter	Median (IQR)/ n (%)
**Age (years)**	84.5 (80.0-91.0)
**Sex**	
Female	34 (56.7%)
Male	26 (43.3%)
**Comorbidities**	
Hypertension	36 (60.0%)
Coronary Artery Disease	20 (33.3%)
Cerebrovascular Disease	14 (23.3%)
Diabetes Mellitus	12 (20.0%)
Congestive Heart Failure	10 (16.7%)
Chronic Obstructive Pulmonary Disease	5 (8.3%)
Chronic Kidney Disease	4 (6.7%)
**Vital Signs**	
Systolic blood pressure, mmHg	115 (97.5-128)
Diastolic blood pressure, mmHg	65.5 (56.5-78.0)
Pulse rate,/min	96.0 (81.0-96.0)
Oxygen saturation, %	92.0 (89.0-95.0)
Respiratory rate,/min	22.0 (19.5-24)
Body temperature, °C	36.7 (36.5-37.0)
**Serum Sodium level (corrected, mmol/L)**	159 (153-169)
**Emergency Department Outcome**	
Admission to the intensive care unit	47 (78.3%)
Admission to the ward	8 (13.3%)
Discharged	2 (3.3%)
Death in the emergency department	3 (5.0%)
**Etiology of hypernatremia**	
Hypovolemic	57 (95.0%)
Hypervolemic	3 (5.0%)
**Mortality**	
24h	3 (5.0%)
30d	27 (45.0%)
**Total**	60 (100.0%)

Values are presented as median (IQR) or n (%). Comorbidities were not mutually exclusive. Patients who died during the index emergency department stay were included in the 24-hour mortality count.

Most patients required hospital admission, with 78.3% admitted to the intensive care unit and 13.3% hospitalized in general wards, while a small proportion were discharged from the emergency department. Hypernatremia was predominantly hypovolemic in etiology (95.0%). Death during the index emergency department stay occurred in 5.0% of patients and was included in the 24-hour mortality count. Overall, 30-day mortality was 45.0%. This high mortality rate likely reflects the advanced age and high comorbidity burden of the study population. Emergency department outcomes, hypernatremia etiology, and mortality data are summarized in Table 1.

### 3.2. Main results

Ultrasonographic parameters changed significantly following fluid resuscitation. The median inferior vena cava (IVC) diameter increased from 1.10 cm (IQR: 0.85–1.45) at baseline to 1.38 cm (IQR: 1.20–1.70) after treatment, corresponding to a median change (ΔIVC) of 0.28 cm (IQR: 0.19–0.35; p < 0.001). In contrast, aortic diameter did not differ significantly between baseline and post-treatment measurements (1.77 cm [IQR: 1.48–2.00] vs. 1.79 cm [IQR: 1.48–2.02]; p = 0.727). The IVC-to-aorta (IVC/Ao) ratio increased from 0.62 (IQR: 0.47–0.87) to 0.80 (IQR: 0.60–1.04), with a median ΔIVC/Ao of 0.115 (IQR: 0.07–0.19; p < 0.001).

Venous pressure measurements also increased following fluid resuscitation. Median peripheral venous pressure rose from 12.5 cm (IQR: 8.0–15.3) to 14.5 cm (IQR: 12.0–18.0), and central venous pressure increased from 5.0 cm (IQR: 4.0–6.0) to 7.0 cm (IQR: 6.0–8.0); both changes were statistically significant (p < 0.001 for each).

Comparisons between patients classified according to early sodium response demonstrated significant between-group differences in ultrasonographic parameters reflecting intravascular volume status (Table 2). Patients without early sodium response had larger IVC diameters at both baseline and post-treatment measurements compared with patients with early sodium response (IVC_1_: p = 0.026, r_rb = −0.366; IVC_2_: p = 0.032, r_rb = −0.353). Similarly, the IVC/Ao ratio was higher in patients without early sodium response at both time points (IVC/Ao_1_: p = 0.037, r_rb = −0.343; IVC/Ao_2_: p = 0.027, r_rb = −0.364). No significant between-group differences were observed for ΔIVC, aortic diameter at either time point, ΔAorta, or ΔIVC/Ao ratio. Peripheral and central venous pressure measurements, including baseline values, post-treatment values, and their changes, were also comparable between groups (all p > 0.30). Effect sizes ranged from negligible to moderate across variables. Baseline calculated serum osmolality was numerically higher in patients with early sodium response than in patients without early sodium response, although this difference did not reach statistical significance (p = 0.052). These findings suggest that factors beyond simple volume depletion may contribute to variability in early sodium correction patterns.

**Table 2:** Comparison of ultrasonographic and manometric measurements between patients with and without early sodium response.

Variable	No early sodium response (n = 42)	Early sodium response (n = 18)	p
*IVC_1_ (cm)*	1.21 (0.88–1.58)	1.00 (0.79–1.10)	**0.026**
*IVC_2_ (cm)*	1.52 (1.24–1.85)	1.32 (1.13–1.39)	**0.032**
*ΔIVC (cm)*	0.28 (0.21–0.36)	0.27 (0.18–0.35)	1.000
*Aorta_1_ (cm)*	1.77 (1.50–1.93)	1.81 (1.38–2.18)	0.561
*Aorta_2_ (cm)*	1.79 (1.51–1.92)	1.80 (1.41–2.19)	0.551
*ΔAorta (cm)*	0.02 (0.01–0.03)	0.02 (–0.03–0.03)	0.403
*IVC/Ao_1_*	0.74 (0.52–0.89)	0.50 (0.43–0.66)	**0.037**
*IVC/Ao_2_*	0.85 (0.63–1.06)	0.66 (0.54–0.81)	**0.027**
*ΔIVC/Ao*	0.12 (0.07–0.21)	0.11 (0.09–0.16)	0.936
*PVP_1_ (cm)*	12.0 (7.3–15.0)	14.0 (10.0–16.8)	0.327
*PVP_2_ (cm)*	13.5 (10.0–17.0)	15.0 (12.0–19.8)	0.323
*ΔPVP (cm)*	2.0 (0.0–4.0)	2.0 (2.0–3.0)	0.922
*CVP_1_ (cm)*	5.0 (4.0–6.0)	5.0 (4.3–6.0)	0.524
*CVP_2_ (cm)*	7.0 (6.0–8.8)	6.5 (6.0–8.0)	0.519
*ΔCVP (cm)*	2.0 (1.3–3.0)	2.0 (1.6–3.0)	0.894
*Osm. (mOsm/kg)*	354 (335-368)	375 (346-384)	0.052

Values are presented as median (IQR). Group comparisons were performed using the Mann–Whitney U test. Δ represents the change between baseline (T1) and 3rd-hour (T2) measurements.

IVC: inferior vena cava diameter; Ao: aortic diameter; PVP: peripheral venous pressure; CVP: central venous pressure; Δ: change between baseline and third-hour measurements; Osm.: calculated osmolality.

**Fig 2:**
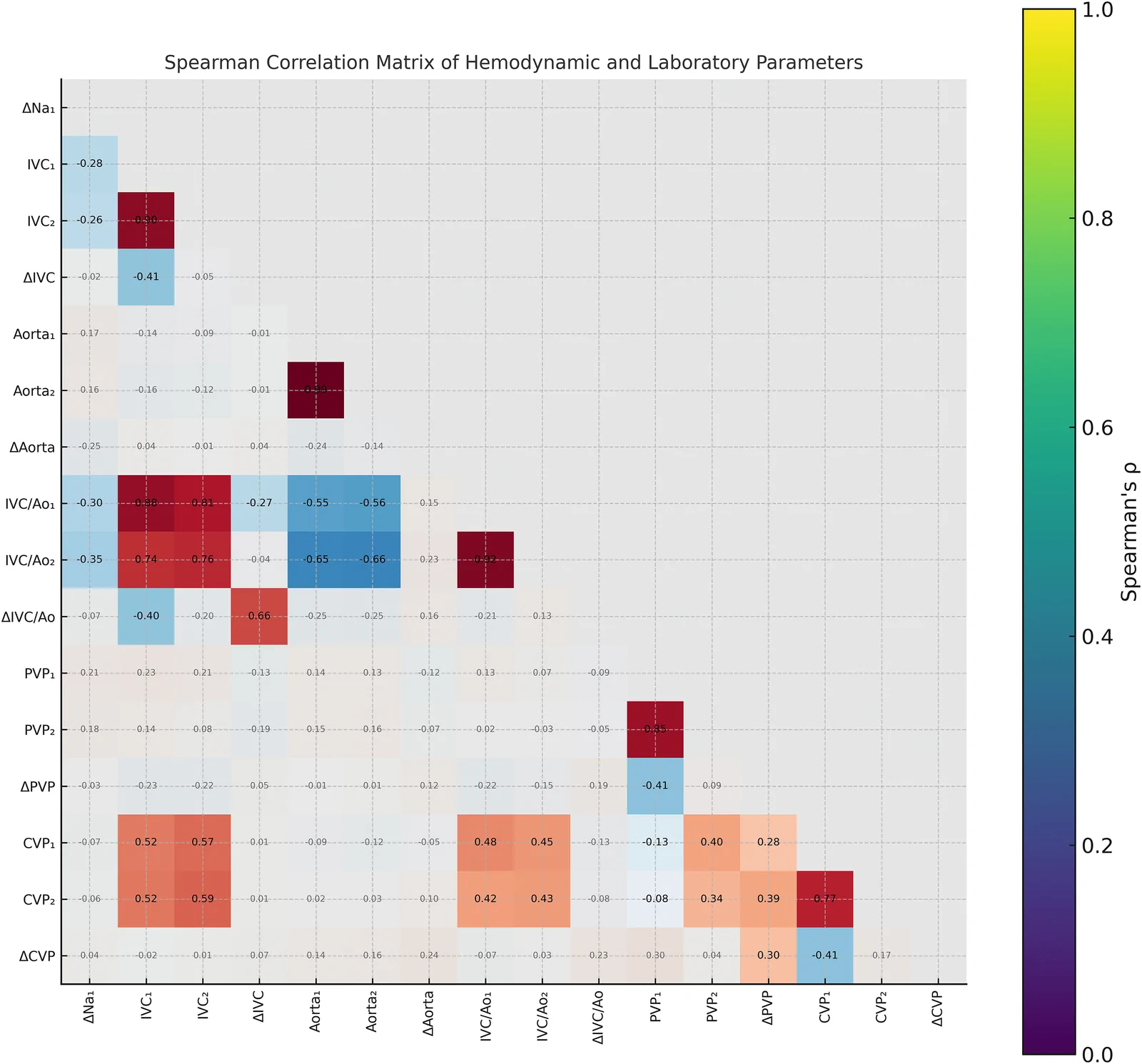
Spearman’s rank correlation heatmap illustrating the relationships among early change in serum sodium (ΔNa_1_), baseline sonographic parameters, and their post-treatment changes. The matrix is displayed in a lower-triangle format. Only statistically significant correlations (p ≤ 0.05) are color-coded and annotated with Spearman’s ρ values. Positive correlations are represented by warmer colors, whereas negative correlations are represented by cooler colors; non-significant associations are left uncolored. Abbreviations: IVC, inferior vena cava diameter; Ao, aortic diameter; PVP, peripheral venous pressure; CVP, central venous pressure. Subscripts 1 and 2 denote baseline and third-hour measurements, respectively.

Associations between early changes in serum sodium (ΔNa_1_) and hemodynamic parameters were evaluated using Spearman’s rank correlation analysis (Fig 2). ΔNa_1_ showed a significant negative correlation with peripheral venous pressure measurements. Moderate negative correlations were also observed between ΔNa_1_ and baseline ultrasonographic indices, including IVC diameter and the IVC/Ao ratio. Strong correlations were noted between repeated measurements of the same parameters, supporting the internal consistency of the measurements. No additional statistically significant correlations were identified.

Early changes in serum sodium were further evaluated in relation to mortality outcomes. Neither the 3-hour (ΔNa_1_) nor the 6-hour (ΔNa_2_) change in serum sodium differed between patients who died within the first 24 hours and those who survived beyond this period (p = 0.814 and p = 0.521, respectively). In contrast, both ΔNa_1_ and ΔNa_2_ were associated with 30-day mortality. Patients who died within 30 days demonstrated smaller reductions in serum sodium at both time points compared with survivors (p = 0.004 and p = 0.010), with moderate effect sizes (r_rb = −0.437 and r_rb = −0.390).

The diagnostic performance of the 3-hour change in serum sodium (ΔNa_1_) in relation to 30-day mortality demonstrated moderate discriminative ability, with an area under the curve of 0.718 (95% CI: 0.593–0.843; p < 0.001). An optimal cut-off value of ≤2.5 mmol/L was identified using Youden’s index, yielding a sensitivity of 92.0% and a specificity of 45.7%.

**Fig 3:**
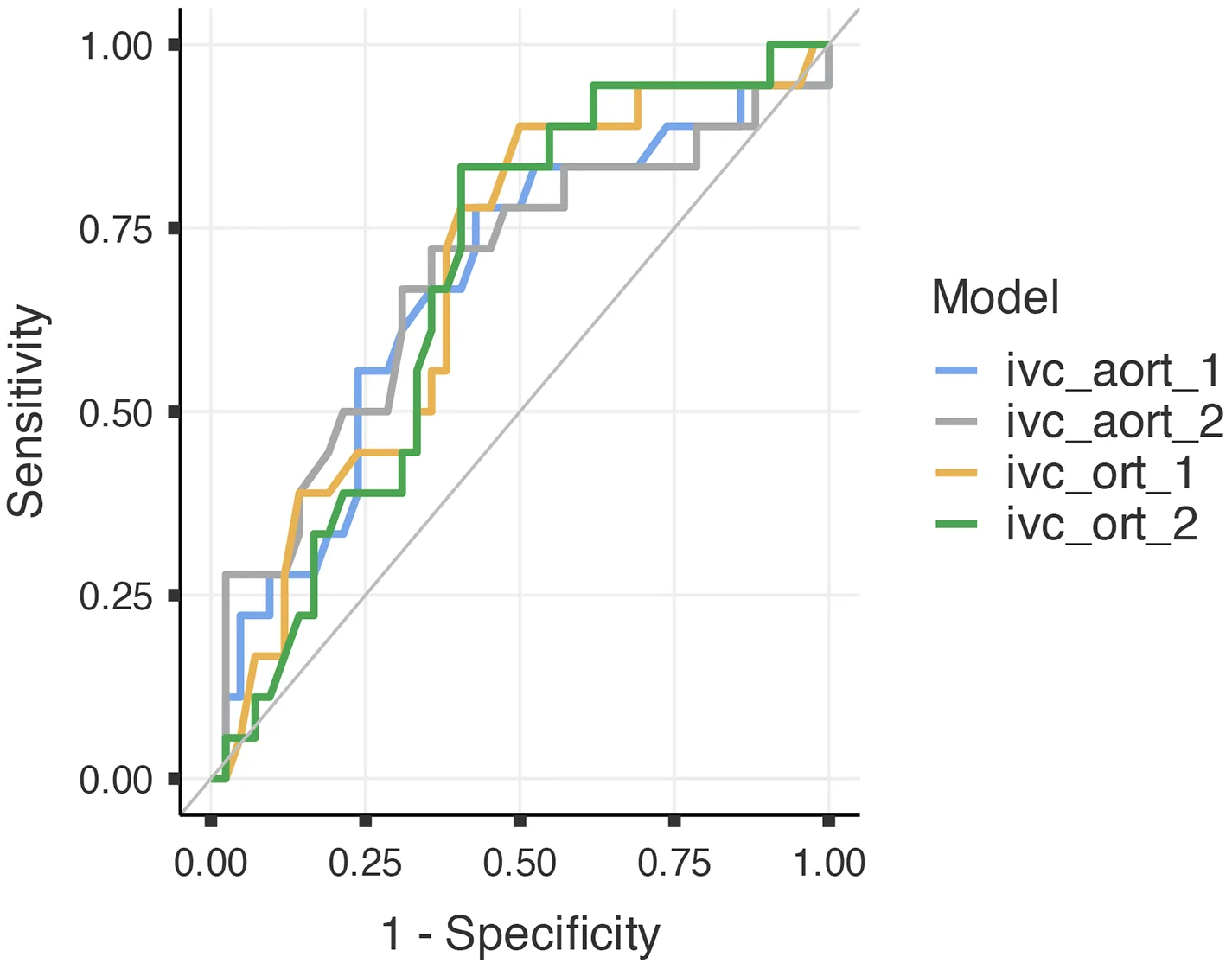
Receiver operating characteristic (ROC) curves of ultrasonographic indices (IVC_1_, IVC_2_, IVC/Ao_1_, IVC/Ao_2_) for predicting early sodium response.

The diagnostic performance of ultrasonographic indices for early sodium response is presented in Table 3 and Fig 3. All evaluated parameters showed moderate discriminative ability, with AUC values ranging from 0.671 to 0.683. Baseline and post-treatment IVC diameter and IVC/Ao ratio measurements demonstrated comparable performance with overlapping confidence intervals. Sensitivity ranged from 72.2% to 88.9%, while specificity ranged from 50.0% to 64.3%. Positive likelihood ratios ranged from 1.78 to 2.06, and negative likelihood ratios were consistently below 0.45. Pairwise comparisons of AUCs using DeLong’s test revealed no statistically significant differences among ultrasonographic parameters (all p > 0.68).

**Table 3:** Diagnostic performance of ultrasound-based indices for predicting early sodium response.

Predictor	AUC (95% CI)	p	Optimal Cut-off*	Sens. %	Spec. %	LR⁺	LR^−^	PPV %	NPV %
IVC_1_ (cm)	0.683 (0.538–0.828)	0.013	≤ 1.215	88.9	50.0	1.78	0.22	43.2	91.3
IVC_2_ (cm)	0.677 (0.535–0.818)	0.014	≤ 1.425	83.3	59.5	2.06	0.28	46.9	89.3
IVC/Ao_1_	0.671 (0.516–0.827)	0.031	≤ 0.675	77.8	57.1	1.82	0.39	43.8	85.7
IVC/Ao_2_	0.682 (0.522–0.842)	0.026	≤ 0.785	72.2	64.3	2.02	0.43	46.4	84.4

*Optimal cut-off values were determined using Youden’s Index.

Sens.: sensitivity; Spec.: specificity; LR⁺: positive likelihood ratio; LR^−^: negative likelihood ratio; PPV: positive predictive value; NPV: negative predictive value.

## 4. Discussion

This study provides an early comparative assessment of ultrasonographic parameters measured during the initial phase of treatment (third hour) in patients presenting to the emergency department with hypernatremia, a condition associated with substantial morbidity and mortality. The findings suggest that baseline and early follow-up measurements of non-invasive ultrasonographic indices, particularly the inferior vena cava–to–aorta (IVC/Ao) ratio, are associated with early sodium response patterns as defined by short-term sodium correction. In contrast, semi-invasive manometric measurements, including peripheral and central venous pressures, did not demonstrate similar discriminatory ability in this cohort.

Following fluid resuscitation, both IVC diameter and the IVC/Ao ratio increased, whereas the aortic diameter remained largely unchanged. This pattern is consistent with the predominant expansion of venous capacitance vessels during fluid administration, while the aorta, a relatively fixed-caliber structure, shows limited short-term dimensional change [7]. Age-related aortic stiffening and calcification, which are common in older populations, may further contribute to the limited variability observed in aortic diameter measurements [10]. In parallel, the IVC/Ao ratio demonstrated a greater ability to differentiate between patients with and without early sodium response compared with manometric measurements, based on both baseline and post-treatment assessments. These findings support the potential utility of ultrasound-derived indices as complementary tools for evaluating intravascular volume status in this patient population.

One of the key findings of this study is the association between baseline intravascular volume status and early sodium response. In the existing literature, no standardized sodium-correction threshold has been established to define early sodium response in patients with hypernatremia, particularly in clinical settings where the duration of hypernatremia cannot be reliably determined. In the absence of consensus regarding early prognostic thresholds, current recommendations for chronic hypernatremia suggest a correction rate not exceeding 10–12 mmol/L over 24 hours, corresponding to approximately 0.5 mmol/L per hour and an expected reduction of around 1.5 mmol/L within the first three hours, whereas more rapid correction has been proposed in acute settings [11]. Although the acute or chronic nature of hypernatremia could not be distinguished in our cohort, these recommendations provide a clinically relevant framework for contextualizing early sodium changes. Accordingly, we derived an exploratory sodium-response threshold by examining the association between early sodium reduction and mortality outcomes. Notably, the identified threshold is broadly consistent with guideline-based expectations for early sodium correction in chronic hypernatremia.

Consistent with established physiological principles, whereby lower IVC/Ao ratios reflect greater degrees of hypovolemia, patients classified as having early sodium response exhibited significantly lower baseline IVC/Ao ratios [12,13]. In contrast, patients without early sodium response demonstrated baseline ratios approaching the proposed threshold for fluid responsiveness (0.675) [13], suggesting a less pronounced intravascular volume deficit. These findings indicate that, in patients with relatively higher baseline IVC/Ao ratios, early sodium correction may not be determined by volume expansion alone, and that additional pathophysiological factors may contribute to variability in early sodium correction patterns. Taken together, despite being a static measurement, the IVC/Ao ratio may offer a practical and non-invasive indicator of effective circulating volume in this clinical context.

In contrast, traditional pressure-based parameters, including central venous pressure (CVP) and peripheral venous pressure (PVP), did not differentiate patients with and without early sodium response at either baseline or after fluid loading. The similarity in median CVP values between groups is consistent with a substantial body of literature questioning the reliability of static venous pressures for estimating intravascular volume status or fluid responsiveness [5,14]. This limitation likely reflects the influence of multiple physiological factors—such as venous compliance, intrathoracic pressure, and right ventricular function—on static pressure measurements, rather than true circulating volume. Although PVP has been shown to correlate with CVP, its lack of discriminatory performance in our cohort further supports the growing shift away from pressure-based monitoring toward volume-oriented assessment strategies [15–17]. Moreover, while the water manometer technique used in this study avoids the need for electronic transducers, its accuracy remains dependent on patient positioning, respiratory variation, and precise identification of the reference point. In this context, the IVC/Ao ratio, which can be obtained rapidly and non-invasively, demonstrated greater discriminatory capacity than manometric measures, supporting its role as a complementary bedside tool for volume assessment in the emergency department.

Another important finding of this study is the association between early sodium correction and subsequent clinical outcomes. A ΔNa_1_ value of ≤2.5 mmol/L within the first three hours was associated with 30-day mortality, with a high negative predictive value indicating that patients with a sodium reduction greater than 2.5 mmol/L were unlikely to die within the 30-day follow-up period. These findings suggest that early sodium change may function as an indicator of early physiologic response patterns and risk stratification rather than a definitive predictor of outcome. Although conventional approaches to hypernatremia management emphasize cautious correction to avoid neurological complications, recent large cohort studies have suggested that early sodium dynamics may carry prognostic information without necessarily supporting more aggressive correction strategies [3,11,18,19]. Importantly, the present findings should be interpreted within a prognostic and observational framework and not as a prescriptive recommendation regarding treatment intensity.

## 5. Limitations

This study has several limitations that should be considered when interpreting the findings. First, the definition of “early sodium response” relied on a sodium-reduction cut-off derived from its association with mortality rather than a universally accepted clinical threshold. Although this exploratory, post-hoc approach requires cautious interpretation, it reflects an attempt to contextualize early biochemical changes in a clinical setting where standardized early response criteria for hypernatremia are lacking.

Second, manometric measurements obtained using a water manometer may not be directly comparable to values derived from catheter-based transducer systems used for invasive central venous pressure monitoring. Nevertheless, this approach reflects real-world practice in many emergency department settings and avoids the risks associated with invasive procedures. Future studies including operators with varying levels of ultrasound experience are needed to further assess generalizability.

Finally, the single-center design and the demographic characteristics of the study population, including advanced age and a high burden of comorbidities, may limit the generalizability of the findings. However, these characteristics also represent a clinically relevant population in which hypernatremia is frequently encountered and associated with adverse outcomes and may therefore reflect real-world emergency department practice.

## 6. Conclusion

In patients presenting to the emergency department with hypernatremia, early ultrasound-based assessment of intravascular volume—particularly using the IVC/Ao ratio—was associated with early sodium response patterns and short-term prognostic outcomes. Higher baseline IVC/Ao ratios were observed in patients with less pronounced early sodium reduction, a pattern that showed an association with increased 30-day mortality. Early changes in serum sodium similarly demonstrated prognostic associations without implying the need for intensified treatment strategies. Together, these findings suggest that non-invasive, volume-oriented hemodynamic assessment may provide complementary physiologic information during the early evaluation of hypernatremia. Integration of ultrasound-based volume assessment with clinical and laboratory evaluation may help improve early bedside assessment strategies in hypernatremic patients. Further large-scale, prospective studies are warranted to validate these observations and to clarify their role within standardized management frameworks.
