# Supplementary material for: Clinical value of the caval–aortic index and inferior vena cava diameter for volume assessment in hypernatremic patients
Source: PLoS One. 2026 Aug 27;21(8):e0356486. doi: 10.1371/journal.pone.0356486 (PMC13521343; doi:10.1371/journal.pone.0356486)
Supplement: Supplementary file 3 [file pone.0356486.s004.docx]

Dear Editor and Reviewers,

We sincerely thank the Editor and the Reviewers for their careful evaluation of our manuscript entitled “Clinical Value of the Caval–Aortic Index and Inferior Vena Cava Diameter for Volume Assessment in Hypernatremic Patients.” We greatly appreciate the insightful and constructive comments provided during the review process.

We have carefully revised the manuscript in accordance with all reviewer suggestions and believe that these revisions have substantially improved the clarity, methodological transparency, and overall scientific quality of the study. In particular, we clarified the exploratory nature of the early sodium-response classification, refined the interpretation of early sodium dynamics, expanded the Discussion section with additional literature comparisons, revised the terminology throughout the manuscript to avoid overinterpretation, and improved the overall language and readability of the text.

In particular, we clarified the exploratory nature of the early sodium-response classification, revised the terminology throughout the manuscript to avoid overinterpretation, refined the interpretation of mortality-related findings, expanded the Discussion and Limitations sections, and strengthened the clinical contextualization of the results.

All reviewer comments are reproduced below in italics, followed by our detailed responses. Changes made in the revised manuscript have been incorporated accordingly.

We again thank the Editor and Reviewers for their valuable time and consideration.

Sincerely,

Tuba Şafak, M.D.

On behalf of all authors

**Reviewer 1**

We sincerely thank the reviewer for the detailed, thoughtful, and constructive evaluation of our manuscript. The comments provided were highly valuable and helped us improve both the methodological clarity and the clinical interpretation of our findings. We carefully considered all concerns raised by the reviewer and revised the manuscript extensively to address these important points. Detailed responses to each comment are provided below.

**Comment 1:**

“...a patient who is responding to fluids will show hemodynamic stability and improvement in the level of consciousness. A change in an electrolyte is not really used during management to assess responsiveness...”

Response:

We sincerely thank the reviewer for this important and thoughtful comment. We fully agree that fluid responsiveness is traditionally assessed using hemodynamic improvement and overall clinical stabilization rather than electrolyte correction alone. We also agree that serum sodium reduction should not be interpreted as a direct surrogate for true hemodynamic responsiveness.

The primary objective of our study, however, was not to establish a standardized definition of fluid responsiveness, but rather to explore the association between early ultrasound-derived intravascular volume indices and short-term biochemical changes during the initial management of hypernatremia. Because hypernatremia management fundamentally aims to achieve gradual sodium correction while restoring volume balance, we considered early sodium dynamics to represent a clinically measurable and objective treatment-related parameter in this specific context.

Importantly, the study population consisted predominantly of very elderly patients with altered mental status, frailty, and multiple comorbidities. In this setting, standardized assessment of clinical responsiveness using neurological improvement or hemodynamic recovery may be difficult to quantify consistently during the early emergency department period. Therefore, we used early sodium change as an exploratory and objective biochemical marker reflecting early treatment dynamics rather than definitive clinical recovery.

In response to the reviewer’s concern, we substantially revised the manuscript to clarify this conceptual framework. Specifically:

We emphasized throughout the manuscript that the response classification was exploratory and outcome-oriented rather than a standardized definition of fluid responsiveness.

We revised the terminology in several sections by replacing expressions such as “treatment responsiveness” with more cautious terms including “early sodium response,” “early biochemical response,” and “early treatment dynamics.”

We further clarified in the Discussion and Limitations sections that sodium correction should be interpreted as a complementary physiological indicator and not as a standalone marker of successful resuscitation or clinical improvement.

We believe these revisions have improved the conceptual clarity of the manuscript and better aligned the interpretation of our findings with current clinical practice.

**Comment 2:**

“I don’t see measuring the IVC/Aorta ratio being more superior to measuring the IVC alone as the article stated the aortic diameter did not change only the IVC diameter changed when the patient is responding to fluids.”

Response:

We thank the reviewer for this insightful comment and agree that the dynamic component primarily reflecting volume change in our study was the inferior vena cava (IVC) diameter, whereas the aortic diameter remained relatively stable throughout the observation period.

We would like to clarify that our intention was not to claim clear superiority of the IVC/Ao ratio over isolated IVC measurement. Rather, the rationale for evaluating the IVC/Ao ratio was based on its potential to normalize IVC measurements against a relatively fixed anatomical structure, thereby possibly reducing interindividual variability related to body habitus and age.

Consistent with the reviewer’s observation, our findings demonstrated that the major treatment-related dimensional change occurred in the IVC itself, while the aortic diameter showed minimal variation. In response to this important concern, we revised several statements throughout the manuscript to avoid overstating the role of the IVC/Ao ratio and to present it more cautiously as a complementary, non-invasive physiologic parameter rather than a superior alternative to isolated IVC assessment.

We additionally expanded the Discussion section to further emphasize that the observed associations were exploratory and should not be interpreted as evidence of superiority over conventional IVC-based evaluation methods.

**Comment 3:**

“The median age of the patients was very high and multiple comorbidities may affect responsiveness to fluids and sodium correction. In addition, the etiology of hypernatremia was broad and underlying causes such as sepsis or heart failure were not sufficiently detailed.”

Response:

We thank the reviewer for this valuable observation and fully agree that advanced age, multiple comorbidities, and the underlying causes of hypernatremia may substantially influence both fluid responsiveness and sodium correction dynamics.

Importantly, the advanced age distribution observed in our cohort is not incidental but rather reflects the well-established epidemiological profile of hypernatremia in emergency and critical care settings. Hypernatremia occurs predominantly in older, frail patients with impaired thirst mechanisms, cognitive dysfunction, limited access to free water, and multiple comorbidities. Therefore, although advanced age and comorbidity burden may introduce clinical heterogeneity, they also represent the real-world population in whom early volume assessment and treatment monitoring are most clinically relevant.

Indeed, the study population consisted predominantly of very elderly patients with a high burden of comorbid disease, reflecting the real-world population in whom hypernatremia is most frequently encountered in emergency department practice. We agree that conditions such as heart failure, renal dysfunction, sepsis, and overall frailty may affect intravascular volume status, treatment response, and short-term sodium kinetics.

In the present study, our primary objective was to evaluate the relationship between ultrasound-derived volume-related indices and early sodium dynamics rather than to investigate disease-specific treatment pathways. Because of the relatively limited sample size and the heterogeneity of underlying conditions, detailed subgroup analyses according to specific etiologies were not statistically reliable. Nevertheless, we reported the predominant hypernatremia classification and the major comorbid conditions of the cohort in Table 1.

In response to the reviewer’s suggestion, we further expanded the Discussion and Limitations sections to more clearly acknowledge that underlying disease processes and comorbidity burden may have influenced sodium correction patterns and overall clinical outcomes. We additionally emphasized that future larger-scale studies focusing on more homogeneous etiological subgroups are needed to better clarify these relationships.

**Comment 4:**

“The secondary aim regarding 30-day mortality was not fully explained, and it may appear that hypernatremia itself was assumed to be the cause of death. It would be better to specify the causes of death.”

Response:

We thank the reviewer for this important comment and agree that mortality in this patient population is multifactorial and should not be attributed solely to hypernatremia or sodium correction dynamics.

The intention of our secondary analysis was not to suggest a direct causal relationship between hypernatremia and mortality, but rather to explore whether early sodium dynamics might be associated with short-term clinical outcomes in a population with severe physiological vulnerability. Given the advanced age, frailty, and substantial comorbidity burden of the cohort, mortality was likely influenced by multiple interacting clinical factors beyond electrolyte disturbance alone.

We agree that detailed adjudication of the exact causes of death would provide additional clinical context. However, because of the observational nature of the study and the complex multimorbid structure of the patient population, standardized determination of primary causes of death was not consistently feasible.

In response to the reviewer’s concern, we revised the manuscript to further clarify that the mortality findings should be interpreted as prognostic associations rather than causal inferences. We additionally emphasized in the Discussion section that early sodium correction dynamics may reflect overall physiological status and treatment response patterns rather than representing an isolated determinant of mortality.

**Comment 5:**

“The authors mentioned comparing central venous pressure and peripheral venous pressure with the IVC/Aorta ratio, but this was not reflected in the title.”

Response:

We thank the reviewer for this thoughtful observation. We agree that the study included comparative evaluation of ultrasonographic and manometric parameters, including central venous pressure and peripheral venous pressure measurements.

However, the primary focus of the study was the clinical utility of ultrasound-based intravascular volume assessment, particularly the IVC diameter and IVC/Ao ratio, in hypernatremic patients. The manometric measurements were incorporated mainly as reference and comparative physiologic parameters rather than as the principal subject of investigation.

For this reason, we preferred to maintain a concise and focused title centered on the ultrasound-based indices. Nevertheless, in response to the reviewer’s suggestion, we revised several sections of the Abstract, Methods, and Discussion to more clearly emphasize the comparative nature of the study design and the inclusion of manometric measurements within the overall analytical framework.

**Final Comment:**

“It was an interesting article to review; however, there are a lot of issues that need to be clarified in order to make the goal of the article stronger.”

Response:

We sincerely thank the reviewer for the comprehensive and constructive evaluation of our manuscript. We carefully considered all comments and substantially revised the manuscript to improve conceptual clarity, methodological transparency, and the interpretation of our findings. In particular, we clarified the exploratory nature of the early sodium-response classification, revised terminology throughout the manuscript to avoid overinterpretation, expanded the Discussion and Limitations sections, and further contextualized our findings within the existing literature. We believe these revisions have significantly strengthened the scientific clarity and overall presentation of the study.

**Reviewer 2**

**Comment 1:**

“Abstract: methodology need more details.”

Response:

We thank the reviewer for this valuable suggestion. In response, we revised the Abstract section to provide additional methodological clarification and improve overall transparency. Specifically, we further emphasized the prospective observational design, the emergency department setting, the timing of ultrasonographic and manometric measurements before and after fluid resuscitation, and the exploratory ROC-based classification approach used for early sodium response assessment.

**Comment 2:**

“Subtitles of introduction: should be removed.”

Response:

We thank the reviewer for this suggestion. In accordance with the reviewer’s recommendation, the subheadings within the Introduction section have been removed to improve readability and ensure a more consistent manuscript structure.

**Comment 3:**

“Conclusion: need more clarification.”

Response:

We thank the reviewer for this valuable comment. In response, we revised the Conclusion section to improve conceptual clarity and avoid potential overinterpretation of the findings. Specifically, we further emphasized the exploratory nature of the observed associations, clarified that ultrasound-based parameters should be interpreted as complementary physiologic indicators rather than standalone determinants of treatment success, and highlighted the need for further large-scale prospective validation studies.

**Comment 4:**

“Methodology: well written but need references for new terms.”

Response:

We thank the reviewer for this helpful suggestion. In response, we carefully reviewed the Methods section and further clarified the terminology and methodological concepts used throughout the manuscript. Relevant references supporting the principal ultrasound-based indices and treatment assessment approaches were already included and have been reviewed for consistency and clarity in the revised version.

**Comment 5:**

“Table 2: abbreviations should be mentioned below table.”

Response:

We thank the reviewer for this helpful comment. In response, the abbreviations used in Table 2 have been clearly defined below the table to improve readability and facilitate interpretation.

**Comment 6:**

“Language need to be revised.”

Response:

We thank the reviewer for this valuable comment. The manuscript has been carefully revised throughout to improve language clarity, readability, grammar, and overall academic style. Particular attention was given to sentence structure, terminology consistency, and the clarity of methodological and discussion sections.

**Comment 7:**

“Discussion: need another papers to be mentioned and compare this study with (agreeing or disagreeing with) and mention causes of agreement or disagreement.”

Response:

We thank the reviewer for this valuable suggestion. In response, we expanded the Discussion section to further contextualize our findings within the existing literature. Additional comparative interpretations were incorporated to better discuss areas of agreement and divergence between our results and previously published studies, particularly regarding ultrasound-based volume assessment and the limitations of pressure-based parameters. Possible physiological and methodological explanations for these similarities and differences were also further clarified in the revised manuscript.

**Comment 8:**

“Recommendations: not mentioned.”

Response:

We thank the reviewer for this helpful suggestion. In response, we expanded the concluding sections of the manuscript to more clearly emphasize the clinical implications of the findings and provide recommendations for future research. Specifically, we highlighted the need for larger prospective multicenter studies, evaluation in more homogeneous patient populations, and further validation of ultrasound-based volume assessment approaches integrated with clinical and laboratory assessment strategies in hypernatremic patients.
