# Supplementary material for: Clinical value of the caval–aortic index and inferior vena cava diameter for volume assessment in hypernatremic patients
Source: PLoS One. 2026 Aug 27;21(8):e0356486. doi: 10.1371/journal.pone.0356486 (PMC13521343; doi:10.1371/journal.pone.0356486)
Supplement: Supplementary file 4 [file pone.0356486.s001.docx]

Abstract: methodology need more details

Subtitles of introduction: should be removed

Conclusion : need more clarification

Methodology : well written but need references for new terms

Table (2): abbreviations should be mentioned below table

Language need to be revised

Discussion : need another papers to be mentioned and compare this study with (agreeing or disagreeing with) and mention causes of agreement or disagreement

Recommendations : not mentioned
