# Supplementary material for: Clinical value of the caval–aortic index and inferior vena cava diameter for volume assessment in hypernatremic patients
Source: PLoS One. 2026 Aug 27;21(8):e0356486. doi: 10.1371/journal.pone.0356486 (PMC13521343; doi:10.1371/journal.pone.0356486)
Supplement: Supplementary file 5 [file pone.0356486.s002.docx]

Reviewing the article “Clinical Value of the Caval-Aortic Index and Inferior Vena Cava Diameter for Volume Assessment in Hypernatremic Patients”

While reading the article, the authors mentioned that they were comparing central venous pressure and peripheral pressure with that of the IVC/Aorta ratio to see if it was compatible, but this was not mentioned in the title. The other thing is that, in order to see a response, there has to be a short-term change in serum sodium levels which is the goal of this article. In my opinion, a patient who is responding to fluids will show hemodynamic stability and improvement in the level of consciousness. A change in an electrolyte is not really used during management to assess responsiveness. If the patient has high potassium with ECG changes or has acidosis and when you give fluids and see improvement, then this may be used as responsiveness to your management but not by using sodium level alone.

There is a lot of research on checking IVC for fluid responsiveness. When the IVC is collapsed, fluid management is started, then the IVC is checked is there is any change in diameter and also hemodynamic stability of the patient which will assess responsiveness. I don’t see measuring the IVC/Aorta ratio being more superior to measuring the IVC alone as the article stated the aortic diameter did not change only the IVC diameter changed when the patient is responding to fluids.

When looking at table 1 in the article, it states that the median age of the patients participating in the study is 84 years old. This means that there will be multiple comorbidity which will affect the patient responsiveness to fluid and their ability to decrease their sodium levels. In addition, the etiology of hypernatremia was broad, for example hypo/hypervolemia. These are broad terms and usually have an underlying cause, for example sepsis or heart failure. It would be more convenient to put the causes of hypo/hypervolemia as hypernatremia is caused by an underlying disease meaning hypovolemia hypervolemia secondary to sepsis as this will change in seeing if the patient really responds to fluids or not. Also, patients with heart failure or renal failure will require different management and this will affect the responsiveness of the patient and in turn cause a decrease in the serum sodium.

The secondary aim of the article is to see the 30-day mortality in patients who did not have a decrease in the sodium level with the resuscitative timeline which caused them to die. This was not explained in full details as the cause of death at 30 days was anticipated to be due to the hypernatremia which is not the issue. It would be better to put the cause of death of these patients to see if the hypernatremia was really the cause.

It was an interesting article to review, however there are a lot of issues that need to be clarified in order to make goal of the article more stronger.
