# Supplementary material for: Clinical value of the caval–aortic index and inferior vena cava diameter for volume assessment in hypernatremic patients
Source: PLoS One. 2026 Aug 27;21(8):e0356486. doi: 10.1371/journal.pone.0356486 (PMC13521343; doi:10.1371/journal.pone.0356486)
Supplement: Peer review history

# Peer review: Decision Letter 0

**DOI:** 10.1371/journal.pone.0356486.r001

**Authors:** Yusuke Hoshino

21 May 2026

Dear Dr. Safak,

Thank you for submitting your manuscript to PLOS ONE. After careful consideration, we feel that it has merit but does not fully meet PLOS ONE’s publication criteria as it currently stands. Therefore, we invite you to submit a revised version of the manuscript that addresses the points raised during the review process.

Please submit your revised manuscript by Jul 05 2026 11:59PM. If you will need more time than this to complete your revisions, please reply to this message or contact the journal office at plosone@plos.org. When you're ready to submit your revision, log on to https://www.editorialmanager.com/pone/ and select the 'Submissions Needing Revision' folder to locate your manuscript file.

- A letter that responds to each point raised by the academic editor and reviewer(s). You should upload this letter as a separate file labeled 'Response to Reviewers'.
- A marked-up copy of your manuscript that highlights changes made to the original version. You should upload this as a separate file labeled 'Revised Manuscript with Track Changes'.
- An unmarked version of your revised paper without tracked changes. You should upload this as a separate file labeled 'Manuscript'.

If you would like to make changes to your financial disclosure, please include your updated statement in your cover letter. Guidelines for resubmitting your figure files are available below the reviewer comments at the end of this letter.

If applicable, we recommend that you deposit your laboratory protocols in protocols.io to enhance the reproducibility of your results. Protocols.io assigns your protocol its own identifier (DOI) so that it can be cited independently in the future. For instructions see: https://journals.plos.org/plosone/s/submission-guidelines#loc-laboratory-protocols. Additionally, PLOS ONE offers an option for publishing peer-reviewed Lab Protocol articles, which describe protocols hosted on protocols.io. Read more information on sharing protocols at https://plos.org/protocols?utm_medium=editorial-email&utm_source=authorletters&utm_campaign=protocols.

As the corresponding author, your ORCID iD is verified in the submission system and will appear in the published article. PLOS supports the use of ORCID, and we encourage all coauthors to register for an ORCID iD and use it as well. Please encourage your coauthors to verify their ORCID iD within the submission system before final acceptance, as unverified ORCID iDs will not appear in the published article. *Only*  the individual author can complete the verification step; PLOS staff *cannot*  verify ORCID iDs on behalf of authors.

We look forward to receiving your revised manuscript.

Kind regards,

Yusuke Hoshino

Academic Editor

PLOS One

Journal Requirements:

When submitting your revision, we need you to address these additional requirements.

1. Please ensure that your manuscript meets PLOS ONE's style requirements, including those for file naming. The PLOS ONE style templates can be found at

https://journals.plos.org/plosone/s/file?id=wjVg/PLOSOne_formatting_sample_main_body.pdf and

https://journals.plos.org/plosone/s/file?id=ba62/PLOSOne_formatting_sample_title_authors_affiliations.pdf

2. In this instance it seems there may be acceptable restrictions in place that prevent the public sharing of your minimal data. However, in line with our goal of ensuring long-term data availability to all interested researchers, PLOS’ Data Policy states that authors cannot be the sole named individuals responsible for ensuring data access (http://journals.plos.org/plosone/s/data-availability#loc-acceptable-data-sharing-methods).

Data requests to a non-author institutional point of contact, such as a data access or ethics committee, helps guarantee long term stability and availability of data. Providing interested researchers with a durable point of contact ensures data will be accessible even if an author changes email addresses, institutions, or becomes unavailable to answer requests.

Before we proceed with your manuscript, please also provide non-author contact information (phone/email/hyperlink) for a data access committee, ethics committee, or other institutional body to which data requests may be sent. If no institutional body is available to respond to requests for your minimal data, please consider if there any institutional representatives who did not collaborate in the study, and are not listed as authors on the manuscript, who would be able to hold the data and respond to external requests for data access? If so, please provide their contact information (i.e., email address). Please also provide details on how you will ensure persistent or long-term data storage and availability.

3. Please include captions for your Supporting Information files at the end of your manuscript, and update any in-text citations to match accordingly. Please see our Supporting Information guidelines for more information: http://journals.plos.org/plosone/s/supporting-information.

4. If the reviewer comments include a recommendation to cite specific previously published works, please review and evaluate these publications to determine whether they are relevant and should be cited. There is no requirement to cite these works unless the editor has indicated otherwise.

[Note: HTML markup is below. Please do not edit.]

Reviewers' comments:

Reviewer's Responses to Questions

**Comments to the Author**

1. Is the manuscript technically sound, and do the data support the conclusions?

Reviewer #1: Yes

Reviewer #2: No

**********

2. Has the statistical analysis been performed appropriately and rigorously?

Reviewer #1: Yes

Reviewer #2: No

**********

3. Have the authors made all data underlying the findings in their manuscript fully available?

The PLOS Data policy

Reviewer #1: Yes

Reviewer #2: No

**********

4. Is the manuscript presented in an intelligible fashion and written in standard English?

Reviewer #1: Yes

Reviewer #2: Yes

**********

Reviewer #1: Abstract: methodology need more details

Subtitles of introduction should be removed

Conclusion: need more clarification

Methodology: well written but need references for new terms

Table (2): abbreviations should be mentioned below the table.

Language need to be revised

Discussion: need another papers to be mentioned and compare this study with (agreeing or disagreeing with) and mention causes of agreement or disagreement

Recommendations: not mentioned

Reviewer #2: Reviewing the article “Clinical Value of the Caval-Aortic Index and Inferior Vena Cava Diameter for Volume Assessment in Hypernatremic Patients”

While reading the article, the authors mentioned that they were comparing central venous pressure and peripheral pressure with that of the IVC/Aorta ratio to see if it was compatible, but this was not mentioned in the title. The other thing is that, in order to see a response, there has to be a short-term change in serum sodium levels which is the goal of this article. In my opinion, a patient who is responding to fluids will show hemodynamic stability and improvement in the level of consciousness. A change in an electrolyte is not really used during management to assess responsiveness. If the patient has high potassium with ECG changes or has acidosis and when you give fluids and see improvement, then this may be used as responsiveness to your management but not by using sodium level alone.

There is a lot of research on checking IVC for fluid responsiveness. When the IVC is collapsed, fluid management is started, then the IVC is checked is there is any change in diameter and also hemodynamic stability of the patient which will assess responsiveness. I don’t see measuring the IVC/Aorta ratio being more superior to measuring the IVC alone as the article stated the aortic diameter did not change only the IVC diameter changed when the patient is responding to fluids.

When looking at table 1 in the article, it states that the median age of the patients participating in the study is 84 years old. This means that there will be multiple comorbidity which will affect the patient responsiveness to fluid and their ability to decrease their sodium levels. In addition, the etiology of hypernatremia was broad, for example hypo/hypervolemia. These are broad terms and usually have an underlying cause, for example sepsis or heart failure. It would be more convenient to put the causes of hypo/hypervolemia as hypernatremia is caused by an underlying disease meaning hypovolemia hypervolemia secondary to sepsis as this will change in seeing if the patient really responds to fluids or not. Also, patients with heart failure or renal failure will require different management and this will affect the responsiveness of the patient and in turn cause a decrease in the serum sodium.

The secondary aim of the article is to see the 30-day mortality in patients who did not have a decrease in the sodium level with the resuscitative timeline which caused them to die. This was not explained in full details as the cause of death at 30 days was anticipated to be due to the hypernatremia which is not the issue. It would be better to put the cause of death of these patients to see if the hypernatremia was really the cause.

It was an interesting article to review, however there are a lot of issues that need to be clarified in order to make goal of the article more stronger.

**********

what does this mean?). If published, this will include your full peer review and any attached files.

If you choose “no”, your identity will remain anonymous but your review may still be made public.

**Do you want your identity to be public for this peer review?**  For information about this choice, including consent withdrawal, please see our Privacy Policy

Reviewer #1: No

Reviewer #2: No

**********

[NOTE: If reviewer comments were submitted as an attachment file, they will be attached to this email and accessible via the submission site. Please log into your account, locate the manuscript record, and check for the action link "View Attachments". If this link does not appear, there are no attachment files.]

To ensure your figures meet our technical requirements, please review our figure guidelines: https://journals.plos.org/plosone/s/figures

You may also use PLOS’s free figure tool, NAAS, to help you prepare publication quality figures: https://journals.plos.org/plosone/s/figures#loc-tools-for-figure-preparation.

NAAS will assess whether your figures meet our technical requirements by comparing each figure against our figure specifications.

Attachment Submitted filename: reviewer 2026 plos.docx

Attachment Submitted filename: Reviewing the article.docx

# Author comment: Author response to Decision Letter 1

**DOI:** 10.1371/journal.pone.0356486.r002

2 Jun 2026

Dear Editor and Reviewers,

We sincerely thank the Editor and the Reviewers for their careful evaluation of our manuscript entitled “Clinical Value of the Caval–Aortic Index and Inferior Vena Cava Diameter for Volume Assessment in Hypernatremic Patients.” We greatly appreciate the insightful and constructive comments provided during the review process.

We have carefully revised the manuscript in accordance with all reviewer suggestions and believe that these revisions have substantially improved the clarity, methodological transparency, and overall scientific quality of the study. In particular, we clarified the exploratory nature of the early sodium-response classification, refined the interpretation of early sodium dynamics, expanded the Discussion section with additional literature comparisons, revised the terminology throughout the manuscript to avoid overinterpretation, and improved the overall language and readability of the text.

In particular, we clarified the exploratory nature of the early sodium-response classification, revised the terminology throughout the manuscript to avoid overinterpretation, refined the interpretation of mortality-related findings, expanded the Discussion and Limitations sections, and strengthened the clinical contextualization of the results.

All reviewer comments are reproduced below in italics, followed by our detailed responses. Changes made in the revised manuscript have been incorporated accordingly.

We again thank the Editor and Reviewers for their valuable time and consideration.

Sincerely,

Tuba Şafak, M.D.

On behalf of all authors

Reviewer 1

We sincerely thank the reviewer for the detailed, thoughtful, and constructive evaluation of our manuscript. The comments provided were highly valuable and helped us improve both the methodological clarity and the clinical interpretation of our findings. We carefully considered all concerns raised by the reviewer and revised the manuscript extensively to address these important points. Detailed responses to each comment are provided below.

Comment 1:

“...a patient who is responding to fluids will show hemodynamic stability and improvement in the level of consciousness. A change in an electrolyte is not really used during management to assess responsiveness...”

Response:

We sincerely thank the reviewer for this important and thoughtful comment. We fully agree that fluid responsiveness is traditionally assessed using hemodynamic improvement and overall clinical stabilization rather than electrolyte correction alone. We also agree that serum sodium reduction should not be interpreted as a direct surrogate for true hemodynamic responsiveness.

The primary objective of our study, however, was not to establish a standardized definition of fluid responsiveness, but rather to explore the association between early ultrasound-derived intravascular volume indices and short-term biochemical changes during the initial management of hypernatremia. Because hypernatremia management fundamentally aims to achieve gradual sodium correction while restoring volume balance, we considered early sodium dynamics to represent a clinically measurable and objective treatment-related parameter in this specific context.

Importantly, the study population consisted predominantly of very elderly patients with altered mental status, frailty, and multiple comorbidities. In this setting, standardized assessment of clinical responsiveness using neurological improvement or hemodynamic recovery may be difficult to quantify consistently during the early emergency department period. Therefore, we used early sodium change as an exploratory and objective biochemical marker reflecting early treatment dynamics rather than definitive clinical recovery.

In response to the reviewer’s concern, we substantially revised the manuscript to clarify this conceptual framework. Specifically:

We emphasized throughout the manuscript that the response classification was exploratory and outcome-oriented rather than a standardized definition of fluid responsiveness.

We revised the terminology in several sections by replacing expressions such as “treatment responsiveness” with more cautious terms including “early sodium response,” “early biochemical response,” and “early treatment dynamics.”

We further clarified in the Discussion and Limitations sections that sodium correction should be interpreted as a complementary physiological indicator and not as a standalone marker of successful resuscitation or clinical improvement.

We believe these revisions have improved the conceptual clarity of the manuscript and better aligned the interpretation of our findings with current clinical practice.

Comment 2:

“I don’t see measuring the IVC/Aorta ratio being more superior to measuring the IVC alone as the article stated the aortic diameter did not change only the IVC diameter changed when the patient is responding to fluids.”

Response:

We thank the reviewer for this insightful comment and agree that the dynamic component primarily reflecting volume change in our study was the inferior vena cava (IVC) diameter, whereas the aortic diameter remained relatively stable throughout the observation period.

We would like to clarify that our intention was not to claim clear superiority of the IVC/Ao ratio over isolated IVC measurement. Rather, the rationale for evaluating the IVC/Ao ratio was based on its potential to normalize IVC measurements against a relatively fixed anatomical structure, thereby possibly reducing interindividual variability related to body habitus and age.

Consistent with the reviewer’s observation, our findings demonstrated that the major treatment-related dimensional change occurred in the IVC itself, while the aortic diameter showed minimal variation. In response to this important concern, we revised several statements throughout the manuscript to avoid overstating the role of the IVC/Ao ratio and to present it more cautiously as a complementary, non-invasive physiologic parameter rather than a superior alternative to isolated IVC assessment.

We additionally expanded the Discussion section to further emphasize that the observed associations were exploratory and should not be interpreted as evidence of superiority over conventional IVC-based evaluation methods.

Comment 3:

“The median age of the patients was very high and multiple comorbidities may affect responsiveness to fluids and sodium correction. In addition, the etiology of hypernatremia was broad and underlying causes such as sepsis or heart failure were not sufficiently detailed.”

Response:

We thank the reviewer for this valuable observation and fully agree that advanced age, multiple comorbidities, and the underlying causes of hypernatremia may substantially influence both fluid responsiveness and sodium correction dynamics.

Importantly, the advanced age distribution observed in our cohort is not incidental but rather reflects the well-established epidemiological profile of hypernatremia in emergency and critical care settings. Hypernatremia occurs predominantly in older, frail patients with impaired thirst mechanisms, cognitive dysfunction, limited access to free water, and multiple comorbidities. Therefore, although advanced age and comorbidity burden may introduce clinical heterogeneity, they also represent the real-world population in whom early volume assessment and treatment monitoring are most clinically relevant.

Indeed, the study population consisted predominantly of very elderly patients with a high burden of comorbid disease, reflecting the real-world population in whom hypernatremia is most frequently encountered in emergency department practice. We agree that conditions such as heart failure, renal dysfunction, sepsis, and overall frailty may affect intravascular volume status, treatment response, and short-term sodium kinetics.

In the present study, our primary objective was to evaluate the relationship between ultrasound-derived volume-related indices and early sodium dynamics rather than to investigate disease-specific treatment pathways. Because of the relatively limited sample size and the heterogeneity of underlying conditions, detailed subgroup analyses according to specific etiologies were not statistically reliable. Nevertheless, we reported the predominant hypernatremia classification and the major comorbid conditions of the cohort in Table 1.

In response to the reviewer’s suggestion, we further expanded the Discussion and Limitations sections to more clearly acknowledge that underlying disease processes and comorbidity burden may have influenced sodium correction patterns and overall clinical outcomes. We additionally emphasized that future larger-scale studies focusing on more homogeneous etiological subgroups are needed to better clarify these relationships.

Comment 4:

“The secondary aim regarding 30-day mortality was not fully explained, and it may appear that hypernatremia itself was assumed to be the cause of death. It would be better to specify the causes of death.”

Response:

We thank the reviewer for this important comment and agree that mortality in this patient population is multifactorial and should not be attributed solely to hypernatremia or sodium correction dynamics.

The intention of our secondary analysis was not to suggest a direct causal relationship between hypernatremia and mortality, but rather to explore whether early sodium dynamics might be associated with short-term clinical outcomes in a population with severe physiological vulnerability. Given the advanced age, frailty, and substantial comorbidity burden of the cohort, mortality was likely influenced by multiple interacting clinical factors beyond electrolyte disturbance alone.

We agree that detailed adjudication of the exact causes of death would provide additional clinical context. However, because of the observational nature of the study and the complex multimorbid structure of the patient population, standardized determination of primary causes of death was not consistently feasible.

In response to the reviewer’s concern, we revised the manuscript to further clarify that the mortality findings should be interpreted as prognostic associations rather than causal inferences. We additionally emphasized in the Discussion section that early sodium correction dynamics may reflect overall physiological status and treatment response patterns rather than representing an isolated determinant of mortality.

Comment 5:

“The authors mentioned comparing central venous pressure and peripheral venous pressure with the IVC/Aorta ratio, but this was not reflected in the title.”

Response:

We thank the reviewer for this thoughtful observation. We agree that the study included comparative evaluation of ultrasonographic and manometric parameters, including central venous pressure and peripheral venous pressure measurements.

However, the primary focus of the study was the clinical utility of ultrasound-based intravascular volume assessment, particularly the IVC diameter and IVC/Ao ratio, in hypernatremic patients. The manometric measurements were incorporated mainly as reference and comparative physiologic parameters rather than as the principal subject of investigation.

For this reason, we preferred to maintain a concise and focused title centered on the ultrasound-based indices. Nevertheless, in response to the reviewer’s suggestion, we revised several sections of the Abstract, Methods, and Discussion to more clearly emphasize the comparative nature of the study design and the inclusion of manometric measurements within the overall analytical framework.

Final Comment:

“It was an interesting article to review; however, there are a lot of issues that need to be clarified in order to make the goal of the article stronger.”

Response:

We sincerely thank the reviewer for the comprehensive and constructive evaluation of our manuscript. We carefully considered all comments and substantially revised the manuscript to improve conceptual clarity, methodological transparency, and the interpretation of our findings. In particular, we clarified the exploratory nature of the early sodium-response classification, revised terminology throughout the manuscript to avoid overinterpretation, expanded the Discussion and Limitations sections, and further contextualized our findings within the existing literature. We believe these revisions have significantly strengthened the scientific clarity and overall presentation of the study.

Reviewer 2

Comment 1:

“Abstract: methodology need more details.”

Response:

We thank the reviewer for this valuable suggestion. In response, we revised the Abstract section to provide additional methodological clarification and improve overall transparency. Specifically, we further emphasized the prospective observational design, the emergency department setting, the timing of ultrasonographic and manometric measurements before and after fluid resuscitation, and the exploratory ROC-based classification approach used for early sodium response assessment.

Comment 2:

“Subtitles of introduction: should be removed.”

Response:

We thank the reviewer for this suggestion. In accordance with the reviewer’s recommendation, the subheadings within the Introduction section have been removed to improve readability and ensure a more consistent manuscript structure.

Comment 3:

“Conclusion: need more clarification.”

Response:

We thank the reviewer for this valuable comment. In response, we revised the Conclusion section to improve conceptual clarity and avoid potential overinterpretation of the findings. Specifically, we further emphasized the exploratory nature of the observed associations, clarified that ultrasound-based parameters should be interpreted as complementary physiologic indicators rather than standalone determinants of treatment success, and highlighted the need for further large-scale prospective validation studies.

Comment 4:

“Methodology: well written but need references for new terms.”

Response:

We thank the reviewer for this helpful suggestion. In response, we carefully reviewed the Methods section and further clarified the terminology and methodological concepts used throughout the manuscript. Relevant references supporting the principal ultrasound-based indices and treatment assessment approaches were already included and have been reviewed for consistency and clarity in the revised version.

Comment 5:

“Table 2: abbreviations should be mentioned below table.”

Response:

We thank the reviewer for this helpful comment. In response, the abbreviations used in Table 2 have been clearly defined below the table to improve readability and facilitate interpretation.

Comment 6:

“Language need to be revised.”

Response:

We thank the reviewer for this valuable comment. The manuscript has been carefully revised throughout to improve language clarity, readability, grammar, and overall academic style. Particular attention was given to sentence structure, terminology consistency, and the clarity of methodological and discussion sections.

Comment 7:

“Discussion: need another papers to be mentioned and compare this study with (agreeing or disagreeing with) and mention causes of agreement or disagreement.”

Response:

We thank the reviewer for this valuable suggestion. In response, we expanded the Discussion section to further contextualize our findings within the existing literature. Additional comparative interpretations were incorporated to better discuss areas of agreement and divergence between our results and previously published studies, particularly regarding ultrasound-based volume assessment and the limitations of pressure-based parameters. Possible physiological and methodological explanations for these similarities and differences were also further clarified in the revised manuscript.

Comment 8:

“Recommendations: not mentioned.”

Response:

We thank the reviewer for this helpful suggestion. In response, we expanded the concluding sections of the manuscript to more clearly emphasize the clinical implications of the findings and provide recommendations for future research. Specifically, we highlighted the need for larger prospective multicenter studies, evaluation in more homogeneous patient populations, and further validation of ult

Attachment Submitted filename: revision letter.docx

# Peer review: Decision Letter 1

**DOI:** 10.1371/journal.pone.0356486.r003

**Authors:** Yusuke Hoshino

5 Aug 2026

Clinical Value of the Caval–Aortic Index and Inferior Vena Cava Diameter for Volume Assessment in Hypernatremic Patients

PONE-D-26-04941R1

Dear Dr. Tuba Safak,

We’re pleased to inform you that your manuscript has been judged scientifically suitable for publication and will be formally accepted for publication once it meets all outstanding technical requirements.

Within one week, you’ll receive an e-mail detailing the required amendments. When these have been addressed, you’ll receive a formal acceptance letter and your manuscript will be scheduled for publication.

An invoice will be generated when your article is formally accepted. Please note, if your institution has a publishing partnership with PLOS and your article meets the relevant criteria, all or part of your publication costs will be covered. Please make sure your user information is up-to-date by logging into Editorial Manager at Editorial Manager® and clicking the ‘Update My Information' link at the top of the page. For questions related to billing, please contact billing support.

If your institution or institutions have a press office, please notify them about your upcoming paper to help maximize its impact. If they’ll be preparing press materials, please inform our press team as soon as possible -- no later than 48 hours after receiving the formal acceptance. Your manuscript will remain under strict press embargo until 2 pm Eastern Time on the date of publication. For more information, please contact onepress@plos.org.

Kind regards,

Yusuke Hoshino

Academic Editor

PLOS One

Additional Editor Comments (optional):

Reviewers' comments:

Reviewer's Responses to Questions

**Comments to the Author**

Reviewer #3: (No Response)

**********

2. Is the manuscript technically sound, and do the data support the conclusions?

Reviewer #3: Yes

**********

3. Has the statistical analysis been performed appropriately and rigorously?

Reviewer #3: Yes

**********

4. Have the authors made all data underlying the findings in their manuscript fully available?

The PLOS Data policy

Reviewer #3: Yes

**********

5. Is the manuscript presented in an intelligible fashion and written in standard English?

Reviewer #3: Yes

**********

Reviewer #3: The authors are to be commended on a well-researched and executed study and manuscript. The authors propose a non-invasive ultrasound-derived method to determine volume status for hypernatremic patients presenting in emergency room. By collecting and measuring cross sections of the IVC and Ao, the authors propose a complimentary method for evaluating intravascular volume status along with central and peripheral venous pressures. The authors comprehensively responded to and included edits from previous reviewers, and greatly improved the manuscript. This article should be of interest to many readers who provide patients with acute and emergent care.

**********

what does this mean?). If published, this will include your full peer review and any attached files.

If you choose “no”, your identity will remain anonymous but your review may still be made public.

**Do you want your identity to be public for this peer review?**  For information about this choice, including consent withdrawal, please see our Privacy Policy

Reviewer #3: **Yes:**  John Carney

**********

# Editor report: Acceptance letter

**DOI:** 10.1371/journal.pone.0356486.r004

**Authors:** Yusuke Hoshino

PONE-D-26-04941R1

PLOS One

Dear Dr. Safak,

I'm pleased to inform you that your manuscript has been deemed suitable for publication in PLOS One. Congratulations! Your manuscript is now being handed over to our production team.

At this stage, our production department will prepare your paper for publication. This includes ensuring the following:

* All references, tables, and figures are properly cited

* All relevant supporting information is included in the manuscript submission,

* There are no issues that prevent the paper from being properly typeset

You will receive further instructions from the production team, including instructions on how to review your proof when it is ready. Please keep in mind that we are working through a large volume of accepted articles, so please give us a few days to review your paper and let you know the next and final steps.

Lastly, if your institution or institutions have a press office, please let them know about your upcoming paper now to help maximize its impact. If they'll be preparing press materials, please inform our press team within the next 48 hours. Your manuscript will remain under strict press embargo until 2 pm Eastern Time on the date of publication. For more information, please contact onepress@plos.org.

You will receive an invoice from PLOS for your publication fee after your manuscript has reached the completed accept phase. If you receive an email requesting payment before acceptance or for any other service, this may be a phishing scheme. Learn how to identify phishing emails and protect your accounts at https://explore.plos.org/phishing.

If we can help with anything else, please email us at customercare@plos.org.

Thank you for submitting your work to PLOS One and supporting open access.

Kind regards,

PLOS ONE Editorial Office Staff

on behalf of

Dr. Yusuke Hoshino

Academic Editor

PLOS One
